# Investigation of features of patients in renal transplantation waiting list: Who wants much more of what for renal transplantation?

**DOI:** 10.12669/pjms.294.3720

**Published:** 2013

**Authors:** Isin Cantekin, Husniye Ferah, Mustafa Keles, Erim Gulcan

**Affiliations:** 1 Isin Cantekin, Department of Medical Nursing, Health Sciences Faculty, Meliksah University, Kayseri, Turkey.; 2 Husniye Ferah, Department of Nephrology Dialysis Center, Ataturk University Medical School, Erzurum, Turkey.; 3 Mustafa Keles, Department of Nephrology, Faculty of Medicine, Ataturk University, Erzurum, Turkey.; 4 Erim Gulcan, Department of Nephrology, Faculty of Medicine, Ataturk University, Erzurum, Turkey.

**Keywords:** End-stage renal disease, Kidney transplantation, Sociodemographic characteristics

## Abstract

***Background and Objective:*** Renal transplant is the best form of treatment for most patients with end-stage renal disease (ESRD), because that therapy improves quality of life, prolongs survival, and is cost-effective. The objective of the study being reported was to compare the sociodemographic characteristics and registration status for renal transplantation of ESRD patients in Turkey who were registered for transplant or not.

***Methods: ***The study was conducted between June and September 2012 on patients of several Dialysis Centers. They all were informed in a one on one interview about the risks and benefits of renal transplants; they were also asked to fill out the questionnaires given to them. The study questionnaire was designed with the help of already published reports to include among others the patient’s age, sex, waiting time and educational status.

***Results:*** Patients who had been registered in the cadaver kidney waiting list were aged 43.85±13.48 in the average, with a balanced sex ratio, average dialysis duration 57.30±51.46 months. Of these patients 45 had finished high school, 87 lived in rural areas and 67 had an income equivalent to expenses.

***Conclusion:*** There was a significant difference among the groups depending on the patients’ residence in an urban or rural environment. Such a difference might be following the greater ease of obtaining relevant information in the urban areas.

## INTRODUCTION

Renal transplant is the best form of treatment for most patients with end-stage renal disease (ESRD), because that therapy improves quality of life, prolongs survival, and is cost-effective. In a report published by the World Health Organization, 59 627 renal transplants were performed worldwide in 2008.^1^ During the last years, though, the number of live donors has shown a tendency to diminish, even though the number of patients waiting for a renal transplant remains unchanged.^2^ Another procedure has therefore consisted in organizing pools of cadaver kidneys to be transplanted to those patients in the waiting lists who present the most favorable laboratory findings and the tissue typing most compatible with that of the cadaver.^3^

An additional system, implemented in many countries, is that of keeping a national, general waiting list by consolidating the patient lists at the facility and area level in order to share the available kidneys.^3^ In the case in which a kidney collected in a given area cannot find an ideal transplant candidate, it will be sent to another hospital where a fitting patient, registered in the national system, is waiting.^4^ Kidney transplantation has not yet reached the desired level in our country. Only 152 out of a total 1,475 transplantations (10.3%) performed in our country up to May 1990 had used cadaver kidneys.^5^ On the other hand, cadaver kidneys made up 87.6% of the total in 1989 in the countries participating in the European Dialysis and Transplant Association (EDTA).^6^

Numerous investigators across several countries have studied end-stage renal disease (ESRD) patients awaiting a renal transplant.^2^^,^^7^ Some of these studies have shown a difference with regard to the socioeconomic status of such patients between those living in Mid-eastern as opposed to Western countries.^8^^,^^9^ To the best of the our knowledge, no such study has been performed, however, within the Turkish society, which one of the biggest society-about 75 million- in Europe.

The objective of the study being reported was to compare the sociodemographic characteristics and registration status for renal transplantation of ESRD patients in Turkey who were registered for transplant or not. It will reveal the profile of patients who do not register, offering guidance to governments to plan campaigns on awareness for these patients.

## METHODS

This multicenter study was conducted between June 2012 and September 2012 on patients who had ESRD and received hemodyalises treatment from Medical School Hospital of Ataturk University, the Erzurum Sifa Hospital, the Erzurum Regional Teaching and Research Hospital, and the FMC Private Dialysis Center. The patients who agreed to participate were included. A total of 337 participants were divided in two groups according to registration status for waiting list. Group I patients which included 122 ESRD patients who registered for transplantation and Group II which included 215 ESRD patients who did not register for transplantation waiting list.. The authorizations necessary to conduct the study were obtained. All patients who gave consent to participate in the study were persons treated by the Dialysis Section. They all were informed in a one on one interview about the risks and benefits of renal transplants; they were also asked to fill out the questionnaires given to them. Criteria for exclusion from the study were death.

***Study Questionnaire: ***The study questionnaire was designed with the help of already published reports to include among others the patient’s age, sex, waiting time and educational status.^4^ A pilot questionnaire was first tested in fifteen hemodialysis patients; the first test showed it to be inappropriate for the planned study. The questionnaire, revised in its content and scope, was then retested on the patients, and made definitive after ascertaining that it was sufficiently informative and easy to answer.

***Socio-demographic characteristics: ***All participants were questioned about their age, sex, educational level, socio-economic status, place of residence and dialysis status and the data recorded.

***Statistical Analysis: ***The sociodemographic characteristics were summarized by descriptive statistics and tested by the chi-squared test. The SPSS 15 software package was used to perform statistical description and analysis. The level of significance was set at p<0.05.

## RESULTS

Mean age of the patients who had been registered in the waiting list for translate cadaver kidney were 43.85±13.48. There were a same number of male and female participants with a average 57.30±51.46 months of dialysis duration. Of these patients 45 (13.3%) had finished high school, 87(25.8%) lived in rural areas and 67 (19.8%) had an income equivalent to expenses. Detailed information regarding participant's characteristics are given in Table-I.

**Table-I:** Sociodemographic Characteristics of Patients who were Either Registered or Not Registered to the Cadaver Kidney Waiting List

	*Group- 1* *(Registered)* *n=122*	*Group-2* *(Not Registered)* *n=215*	*P*
Age (years)	43.85±13.48	61.70±14.43	*<0.001*
Sex	122	215	*0.837*
Female	61	105	
Male	61	110	
Time on Dialysis (months)	57.30±51.46	39.1± 43.7	*0.001*
Educational Status	122	215	*0.057*
Illiterate	16	37	
Literate	12	41	
Elementary school	43	75	
High school	45	53	
Higher education	6	9	
*Residence*	122	215	0.010
Rural	35	96	
Urban	87	119	
*Economical Status*	122	215	0.066
Expenses higher than income	45	52	
Income higher than expenses	10	23	
Income equivalent to expenses	67	140	

Mean age of the patients who had been not registered in the waiting list for translate cadaver kidney were 61.70±14.43. Number of the female patients was slightly higher than number of the male in this group. In addition to, mean of the dialyses period were 39.1±43.7 months for these patients. Of these patients 75 (22.2%) had elementary schooling as well as, 119 (35.3%) of lived in urban resident and of 140 (41.5%) had moderate /high income.

## DISCUSSION

Studies published so far show that the number of renal transplantations in our country, in particular that of cadaver kidney transplantations, is still insufficient.^4^ This may be due either to difficulties in identifying appropriate donors or to patient factors. Our study intended to investigate the patient-related characteristics to determine which of these, if any, affect the registration status for a cadaver kidney waiting list.

According to the Turkish Nephrological Association 2008 data, 29.5% of renal transplants had been performed with cadaver kidneys.^9^ This ratio is higher between the ages of 20 and 44. The increased frequency of registration for cadaver kidneys among younger patients in our study is compatible with this observation, 55.3% of these patients were under 30 years age in this study. The study findings are likewise supported by reports of Gaylin et al^7^, Vamos et al^10^, Mariana et al^11^ and Machado et al.^12^

Diabetic nephropathy is the chief probable cause of ESRD with 60% in our patients, followed by hypertensive nephropathy with 25%. It is possible to speculate that this situation may be due to an increasing life expectancy among diabetic and hypertensive patients, itself following an improved adaptation of these patients to their disease and their increased attention to diet, treatment and care. These data is comparable to those reported for the patient population in the United States. The results reported by Vamos et al are similar.^10^ The chief cause indicated by Sever et al as well as Machado et al is glomerulonephritis.^4^^,^^12^

We have reported that the patients who registered as prospective cadaver renal transplant receivers had a longer dialysis duration. It may be speculated here that long-term dialysis treatment creates weariness, thus increasing the chance of getting oneself registered for the cadaver kidney transplant. Sever et al have reported the opposite finding, i.e. that patients with shorter dialysis durations were more likely than others to request a cadaver kidney.^4^

Our study indicated that sex, educational level and socio-economic status were similar in both groups. Under normal conditions, one may hypothesize that both these characteristics may be positively parallel with registration for the cadaver kidney waiting list. Contradicting the published reports, our study could not show the significant role of either factor, given that both groups displayed comparable educational level and socioeconomic status. This finding would suggest that either education or socioeconomic status is not an important factor in determining cadaver kidney transplantation. Vamos et al and Mariana et al report a higher educational and socioeconomic status among patients who were registered in a transplant waiting list.^10^^,^^11^

Our study includes all patients in Eastern Anatolia; all of these patients had been registered for the first time in the cadaver transplant waiting list. The patient populations studied by Vamos et al^10^, Mariana et al^11^ and Sever et al^4^ included patients registering for the second or third time.

There was a significant difference among the groups depending on the patients’ residence in an urban or rural environment. Such a difference might be following the greater ease of obtaining relevant information in the urban areas.

## CONCLUSION

Our study intended to investigate the patient-related characteristics to determine which of these, if any, affect the registration status for a cadaver kidney waiting list. The increased frequency of registration for cadaver kidneys among younger patients in our study is compatible with this observation. Our study includes all patients in Eastern Anatolia; all of these patients had been registered for the first time in the cadaver transplant waiting list.
